# Molecular Detection and Characterization of Goat Isolate of *Taenia hydatigena* in Turkey

**DOI:** 10.1100/2012/962732

**Published:** 2012-03-12

**Authors:** Armagan Erdem Utuk, Fatma Cigdem Piskin

**Affiliations:** Veterinary Control Central Research Institute, Parasitology and Bee Diseases Laboratory, 06020 Ankara, Turkey

## Abstract

The aim of this study was to provide molecular detection and characterization of the goat isolate of *Taenia hydatigena* from Ankara province of Turkey. For this purpose, PCR amplification of small subunit ribosomal RNA (rrnS) and partial sequencing of mitochondrial cytochrome c oxidase subunit 1 (mt-CO1) genes were performed in a one-month-old dead goat. According to rrnS-PCR results, parasites were identified as *Taenia* spp., and partial sequence of mt-CO1 gene was corresponding to *T. hydatigena*. At the end of the study, we concluded that molecular tools can be used to define species of parasites in cases where the key morphologic features cannot be detected. Nucleotide sequence data of Turkish goat isolate of *T. hydatigena* was submitted to GenBank for other researchers interested in this subject. By this study, molecular detection and characterization of *T. hydatigena* was done for the first time in Turkey.

## 1. Introduction

Cysticercosis caused by the metacestode of the taeniid cestodes is a global infection, which has veterinary, medical, and economic importance. The adult stages of the parasites infect canids, while the metacestodes develop in several species of domestic and wild intermediate mammalian hosts where they develop as fluid-filled larvae in tissues [1–3].

*Cysticercus tenuicollis* is the metacestode of canine tapeworm *Taenia hydatigena*, which has been reported in domestic and wild ruminants, pigs, squirrels, and monkeys [1, 2]. Metacestodes are found attached to the omentum, mesentery, and occasionally on the liver surface; however, unusual locations of *C. tenuicollis* have been described as lungs, kidneys, brain, ovaries, uterine tubes, uterus, cervix, and vagina. An aberrant location of *C. tenuicollis* vesicle inside the chorioallantoic membrane of a goat foetus was reported [4]. Pathogenicity of adult parasites is not high for definitive hosts. However, large numbers of developing cysticerci migrate contemporaneously in the liver of intermediate hosts, producing “hepatitis cysticercosa”, a condition whose gross pathology resembles acute fasciolosis and which is often fatal [1, 2, 5].

 Diagnosis of taeniosis and cysticercosis is based on the morphologic and molecular characteristics of the parasites [1, 2, 6, 7]. Number and length of the large and small hooks, number and layers of testes, number of uterine branches, and structure of cirrus sac are important characteristics for morphologic identification [1–3]. Molecular approaches include restriction fragment length polymorphism (RFLP) analysis, PCR-linked RFLP analysis (PCR-RFLP), and direct comparison of PCR-amplified DNA sequences [6–8]. Today, sequence data of mitochondrial NADH dehydrogenase subunit 1 (mt-ND1) and cytochrome c oxidase subunit 1 (mt-CO1) genes of genus *Taenia (T. taeniaeformis, T. hydatigena, T. pisiformis, T. ovis, T. multiceps, T. serialis, T. saginata, T. solium,* and the Asian *Taenia*) is available on GenBank [8, 9].

The aim of this study was to provide molecular detection and characterization of the goat isolate of *T. hydatigena* by PCR amplification of small subunit ribosomal RNA (rrnS) and partial sequencing of mt-CO1 gene in Ankara province of Turkey.

## 2. Materials and Methods

Liver of a one-month-old dead goat was sent to our laboratory for parasitological examination. For this, liver was cut into one cm^3^ pieces and left into warm water for 30 minutes. Then, liver pieces were removed by squeezing, and sediment was examined [1]. 46 cystic samples were collected from the sediment. While ten of them were examined under light microscope, remaining parasites were taken into phosphate-buffered saline (PBS).

Genomic DNA extraction was done from ten randomly selected samples in PBS using DNeasy TM Tissue Kit (Qiagen, Hilden, Germany) by following the manufacturer's instructions. Cest3 (5′-ygaytctttttaggggaaggtgtg-3′) and Cest5 (5′-gcggtgtgtacmtgagctaaac-3′) primer pair was used to amplify partial rrnS of *Taenia *spp. [10]. Furthermore, JB3 (5′-ttttttgggcatcctgaggtttat-3′) and JB4.5 (5′-taaagaaagaacataatgaaaatg-3′) primer pair was used to amplify partial mt-CO1 gene for sequencing [11]. Genomic DNA of an adult parasite morphologically diagnosed as *T. hydatigena* was used as positive control, and distilled water was used as negative control.

PCR conditions were the same for two primer pairs. PCR was carried out in a final volume of 50 *μ*L, containing 25.75 *μ*L DNase, RNase-free sterile distilled water (Biobasic, Inc), 5 *μ*L 10X PCR buffer, 5 *μ*L 25 mM MgCl_2_, 4 *μ*L 1 mM dNTP mix, 2.5 *μ*L of each primer (50 pmol), 5 *μ*L of template DNA (100–200 ng), and 0.25 *μ*L of TaqDNA polymerase (1.25 IU) (MBI Fermentas). The PCR conditions were: 5 min at 95°C (initial denaturation), 35 cycles of 1 min at 95°C, 1 min at 50°C and 1 min at 72°C, and finally 5 min at 72°C (final extension). The PCR products were separated on agarose gels (1.5%), stained with ethidium bromide, visualized and photographed on an UV transilluminator.

The 446 bp region of mt-CO1 gene of one cystic sample was sequenced by a commercial company (Refgen, Ankara, Turkey). The obtained sequences were edited and aligned, using the Bioedit software [12], and then, sequence analysis was undertaken by BLAST algorithms and databases from the National Center for Biotechnology (http://www.ncbi.nlm.nih.gov/).

## 3. Results

**Figure 1:**
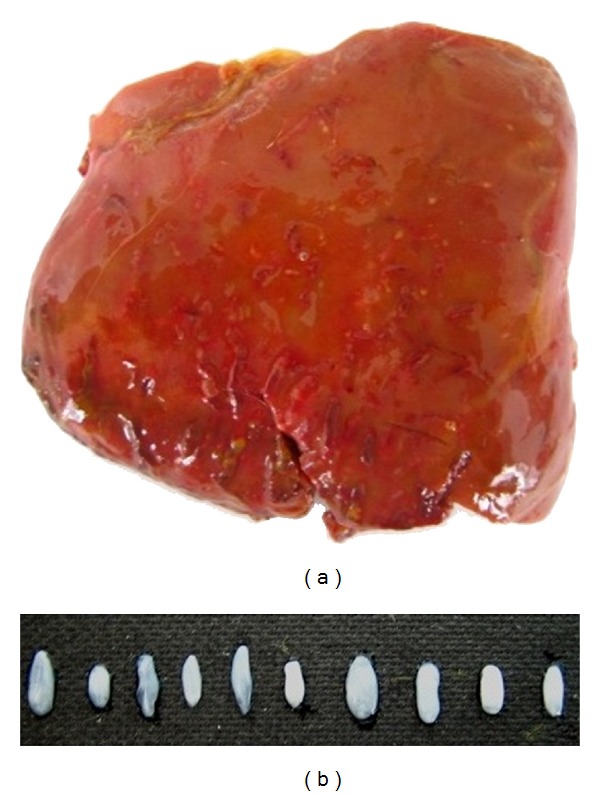
Liver of one-month-old dead goat with haemorrhagic tracts (a) and ten recovered cystic samples (b).

After the macroscopic examination, we detected hemorrhagic tracts and migrating larvae under the liver capsula (Figure 1). In the microscopic examination, we could not detect scoleces, rostellar hooks, or invagination.

**Figure 2:**
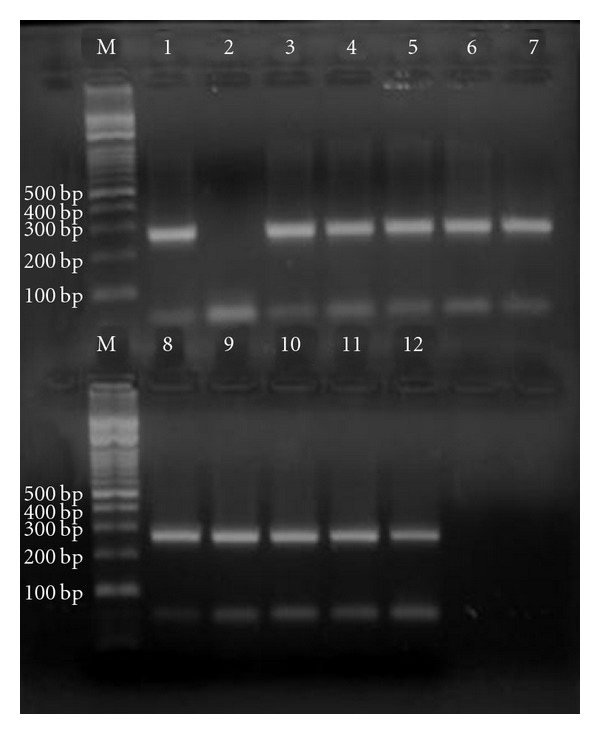
The 267 bp of amplification product of rrnS-PCR with the Cest3 and Cest5 primers M: Marker, 1: Positive control (gDNA of an adult *T. hydatigena*), 2: Negative control (distilled water), 3–12: Ten cystic samples collected from the liver.

**Figure 3:**
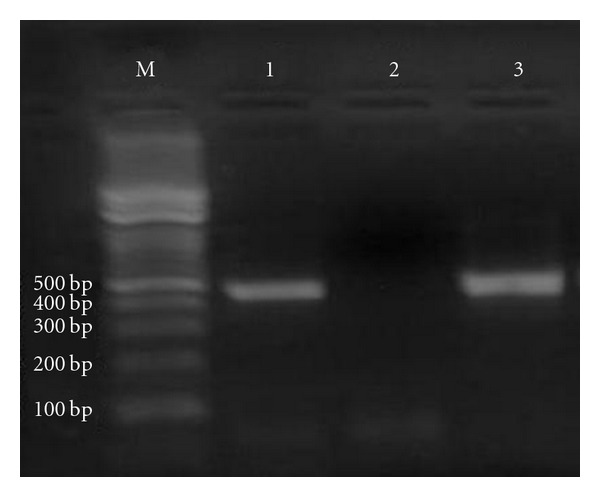
The 446 bp of amplification product of mt-CO1 with the JB3 and JB4.5 primers M: Marker, 1: Positive control (gDNA of an adult *T. hydatigena*), 2: Negative control (distilled water), 3: Cystic sample collected from the liver.

**Figure 4:**
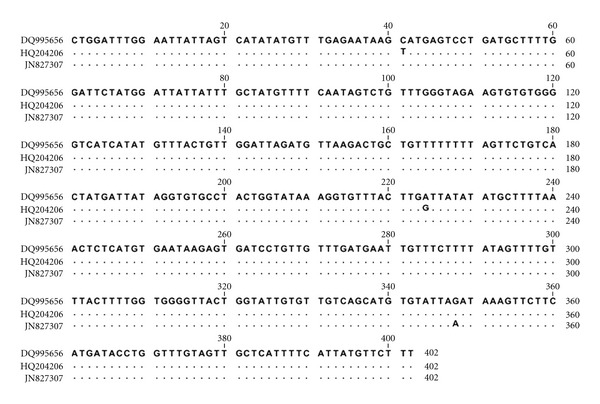
Nucleotide sequences of partial mt-CO1 for *T. hydatigena* analyzed in this study (JN827307) aligned with the published (DQ995656, HQ204206) mt-CO1 sequences of the *T. hydatigena *as a reference. A dot indicates a nucleotide that is conserved relative to the published sequence.

The rrnS-PCR with the Cest3 and Cest5 primers yielded 267 bp of amplification product (Figure 2). According to this, parasites were identified as *Taenia *spp., and mt-CO1-PCR yielded a 446 bp-sized fragment (Figure 3). Partial sequence of mt-CO1 (GenBank accession number: JN827307) was corresponding with *T. hydatigena*. The alignment of the nucleotide sequences with published sequence results (DQ995656, HQ204206) for *T. hydatigena* is presented in Figure 4. According to the alignment results, there was 99% identity in nucleotide sequences for *T. hydatigena.*

## 4. Discussion

*Cysticercus tenuicollis* cysticercosis is a prevalent disease in Turkey. Zeybek [13] found 56.7% prevalence in 252 necropsied lambs. Öge et al. [14] studied on 2484 sheep, 311 goats, and 1941 cattle to determine the prevalence of hydatid cyst, *C. tenuicollis,* and *C. bovis*. According to this study, *C. tenuicollis* was the most prevalent species, and prevalence rates were 26.7% in sheep and 27.9% in goats. Sarımehmetoğlu et al. [15] examined 4931 sheep, 366 cattle, 113 buffaloes, and 112 goats and found 31.8% prevalence in sheep and 28.57% in goats. They could not detect *C. tenuicollis* in cattle and buffaloes. Değer and Biçek [16] studied 220 cattle, 1850 sheep, and 250 goats, and they found the prevalence rates as 8.18%, 65.67%, and 61.60%, respectively. Yıldırım et al. [17] reported acute hepatitis cysticercosa and pneumonitis cysticercosa in a lamb.

In general, it is easy to define the species of *Cysticercus *when the parasite has a certain size and is in specific predilection site; however, in cases of unusual or aberrant localizations, degenerations, and calcifications, species identification and differentiation from cysts of another etiology may be difficult [3]. In Turkey, studies on *C. tenuicollis* focus on the prevalence and postmortem morphologic diagnosis of the disease [13–17]. In this study, we could not detect key morphologic features of larvae such as scoleces, rostellar hooks, or invagination in the microscopic examination. Therefore, we tried to detect *C. tenuicollis* by genus-specific PCR and partial sequencing of mt-CO1 gene. Cest3 and Cest5 primer pair is specific to *Taenia* spp., but not to *Echinococcus* spp. [10]. Thus, this primer pair can be used to differentiate *C. tenuicollis* from the hydatid cysts and other cystic structures. Moreover, partial sequencing of the 446 bp amplicon of JB3 and JB4.5 primer pair enabled us to detect *T. hydatigena* at species level. At the end of the study, we submitted nucleotide sequence data (JN827307) of the Turkish goat isolate of *T. hydatigena* to GenBank for other researchers interested in this subject. We consider that further studies on *T. hydatigena* both in final and in intermediate hosts should be carried out for a clear understanding of the parasite at genetic level.
